# When the algorithm passes the test but fails the patient

**DOI:** 10.1371/journal.pdig.0001774

**Published:** 2026-10-06

**Authors:** Alex Mirugwe

**Affiliations:** Makerere University, School of Public Health, Kampala, Uganda; National Tsing Hua University, TAIWAN

## Abstract

Tens of thousands of AI diagnostic tools have been authorised for clinical diagnosis worldwide, justified primarily by benchmark accuracy (performance on a fixed, curated test dataset), a number that says little about what the tool will do at the bedside. The field rests on three flawed foundations: datasets that do not reflect the populations served, development pipelines that exclude clinicians, and regulatory pathways that mistake on-paper accuracy for evidence of benefit. Closing the gap requires global coordination and the political will to treat clinical validation as a prerequisite rather than an afterthought.

## Introduction

By 2025, tens of thousands of AI medical devices had been authorised worldwide, in a market that industry estimates is valued at roughly USD 13.7 billion [1]. Yet the central claim, that benchmark accuracy translates into clinical benefit, has not been adequately tested. A 2025 npj Digital Medicine meta-analysis of 83 studies of generative AI diagnostic models (specifically large language and multimodal models, not medical AI as a whole) found a pooled accuracy of 52.1% (95% CI 47.0–57.1%), 15.8 percentage points lower than expert physicians (95% CI 4.4–27.1; *p* = 0.007). The included studies were highly heterogeneous, and most carried a high risk of bias, so this pooled figure should be read as an indicator of the evidentiary weakness of this subfield rather than a precise estimate of diagnostic performance [2]. Three structural failures help explain this gap.

## Two cases that illuminate the problem

The Epic Sepsis Model, deployed across health systems worldwide, reportedly identified high-risk patients in 87% of cases. That figure fell to 63% when restricted to data before clinicians suspected sepsis, and to 53% before a blood culture was ordered [3]. The model had learned clinician suspicion, not physiology. In practice, it missed two-thirds of cases and generated so many false alarms that staff at one major system acknowledged only 13% of its alerts in 2023, a figure that reflects alert fatigue and workflow burden as much as model failure [4]. TREWS, developed at Johns Hopkins with sustained input from nurses and intensivists, was instead built around clinical workflow from the start: a prospective multisite trial showed reduced sepsis mortality, shorter organ failure duration, and improved antibiotic timeliness [5,6]. The two systems differ on several dimensions: training data, the definition of the prediction target, deployment context, and institutional support, so the contrast cannot be reduced to a single cause. The clearest methodological difference, however, is that TREWS treated clinical co-design as a technical requirement from the outset rather than as an optional consultation, and this appears to be a major contributing factor in its more favourable real-world performance.

## The dataset problem is a patient safety problem

Representation bias is now a dominant failure mode in AI diagnostic tools [7]. Fewer than 30% of authorised devices disclosed the demographic composition of their training data across a recent decade-long review, though disclosure norms have been shifting over that period [8]; for much of this cohort, clinicians could not know whose biology the algorithm learned from. MIT researchers showed in 2024 that popular diagnostic models on chest radiographs had acquired superhuman ability to predict patient race, sex, and age from images: these models were learning demographic shortcuts that correlated with pathology in the training cohort, correlations that break down elsewhere [9]. A retinopathy algorithm validated at 87% sensitivity delivered 60–80% sensitivity and specificity as low as 14% in public-health settings in northern India, a gap partly attributable to the lower image quality of the non-mydriatic cameras used in routine screening compared with the images used for validation [10]. Same disease, same modality, a different population: a different machine. Tools trained at tertiary academic centres carry case-mix assumptions that do not hold in district hospitals in sub-Saharan Africa or rural Southeast Asia, precisely the settings where AI is promoted as a solution to specialist shortages [11].

## The absent expert in the room

AI diagnostic tools are built by engineers, evaluated by statisticians, and regulated by agencies whose frameworks were designed for drugs and static devices. The clinician who must integrate model output with patient history, examination, and ward context is routinely absent from development [7]. The result is alert fatigue, automation bias, and the silent override in which clinicians learn to ignore unreliable recommendations [4]. Teams combining clinicians and data scientists produce more generalisable models, because clinicians know which features are predictive versus confounded by care patterns [7]. Excluding this knowledge is not a resourcing choice; it is an epistemic error.

## A global regulatory patchwork with the same flaw

Frameworks vary across jurisdictions but share one weakness: they use pre-market accuracy as the gateway to deployment without requiring prospective validation in the populations who will use the tool. In the US, 96.4% of AI medical devices were cleared via 510(k) without clinical trial evidence [12]; of the 950 AI-enabled devices the FDA had authorised through November 2024, 60 were subject to recall (182 recall events in total), and 43% of these recalls occurred within a year of authorisation, mostly for diagnostic errors [13]. The EU AI Act (2024) imposes the most demanding pre-market regime but does not yet specify outcome-level trial requirements [14]. China requires localised trials for Class III devices [8]; India’s 2025 draft guidance classifies AI imaging as Class C, requiring clinical validation [14]; Japan uses an adaptive framework [15]. Across every jurisdiction, demographic disclosure remains the exception rather than the rule, and AI development has outpaced regulation, leaving lower-income settings exposed to tools validated elsewhere [15].

## Toward genuine clinical readiness

Three reforms are achievable. First, prospective external validation on demographically and geographically diverse populations must be a non-negotiable prerequisite for authorisation; the TRIPOD+AI and STARD-AI frameworks already provide the scaffolding. Second, performance must be disaggregated by clinically relevant subgroup. An AI diagnostic tool with 92% overall accuracy but 74% in one subgroup is selectively unreliable, and the patients it fails are predictable [7]; demographic disclosure, still the exception today, should become universal. Third, clinicians must be structural partners in development from problem definition onwards, not consultees on a finished product [5]. Achieving this at scale requires coordination: the International Medical Device Regulators Forum offers the mechanism through which harmonised standards could be adopted across jurisdictions worldwide [15]. These reforms face real obstacles. Much training data is proprietary or retrospective, and privacy-preserving methods such as federated learning can make full demographic disclosure and prospective external validation harder to mandate in practice. These are reasons to phase requirements in, and to develop validation methods suited to distributed and federated data—rather than reasons to keep treating clinical readiness as optional.

## Conclusion

The gap between algorithmic performance and clinical benefit is not an engineering problem awaiting a better model. It is a governance problem created by norms that reward accuracy on curated datasets, pathways that mistake accuracy for clinical readiness, and a publication culture that treats external validation as optional. For the patient in a Lagos clinic whose retinal image is read by an algorithm trained on a different continent, the difference between 87% sensitivity in a trial and 60% in the field is a substantially higher risk of a missed diagnosis [10]. An AI diagnostic tool that passes a benchmark test has not passed a clinical one. Until that distinction is embedded in regulation, publication standards, and funding criteria, the gap will remain a predictable outcome of present incentives rather than an inevitable one. TREWS shows that the gap can be closed where co-design and rigorous validation are taken seriously, and the reforms above are achievable. But until those incentives change, it will persist as the default the current system produces.
